# Annealing Ant Colony Optimization with Mutation Operator for Solving TSP

**DOI:** 10.1155/2016/8932896

**Published:** 2016-11-24

**Authors:** Abdulqader M. Mohsen

**Affiliations:** Department of Computer Science, Faculty of Computing and Information Technology, University of Science and Technology, Sana'a, Yemen

## Abstract

Ant Colony Optimization (ACO) has been successfully applied to solve a wide range of combinatorial optimization problems such as minimum spanning tree, traveling salesman problem, and quadratic assignment problem. Basic ACO has drawbacks of trapping into local minimum and low convergence rate. Simulated annealing (SA) and mutation operator have the jumping ability and global convergence; and local search has the ability to speed up the convergence. Therefore, this paper proposed a hybrid ACO algorithm integrating the advantages of ACO, SA, mutation operator, and local search procedure to solve the traveling salesman problem. The core of algorithm is based on the ACO. SA and mutation operator were used to increase the ants population diversity from time to time and the local search was used to exploit the current search area efficiently. The comparative experiments, using 24 TSP instances from TSPLIB, show that the proposed algorithm outperformed some well-known algorithms in the literature in terms of solution quality.

## 1. Introduction

One of the most popular combinatorial optimization problems is the traveling salesman problem (TSP) [1]. Given a set of cities, a salesman attempts to find the shortest or at least near to the shortest tour by visiting each city only once and turning back to the starting city. TSP is a representative of variety of combinatorial problems. It has been studied for the last 40 years. It has many real world applications such as the movement of people, postal delivery, school bus routes, garbage collection, design of hardware devices and radio electronic systems, machine scheduling, integrated circuits, and computer networks [2–4].

Metaheuristic algorithms are formally defined as algorithms that inspired by nature and biological behaviors. They produce high-quality solutions by applying a robust iterative generation process for exploring and exploiting the search space efficiently and effectively. Recently, metaheuristic algorithms seem to be a hot and promising research areas [5]. They can be applied to find near-optimal solutions in a reasonable time for different combinatorial optimization problems [6].

Metaheuristic algorithms such as genetic algorithms (GAs) [7], particle swarm optimization (PSO) [8], tabu search (TS) [7], simulated annealing (SA) [9], and ant colony optimizations (ACO) [10] are widely used for solving the TSP. Ant colony optimization proposed by Dorigo et al. in 1996 [10] simulates the intelligent behavior of real ants seeking for the food in nature. It has been successfully applied to solve many optimization problems such as TSP [10], quadratic assignment [11], job-shop scheduling [12], and load balancing in telecommunications networks [13].

In applying standalone metaheuristic algorithms, there is possibility of losing the diversity of the population through premature convergence and thus the algorithm gets stuck in local optima. Therefore, maintaining the diversity and making tradeoff between diversification and intensification by combining two or more algorithms to produce high-quality solutions and speed up the execution time is indispensable [14].

For hybrid ACO, the earliest study was conducted by McKendall and Shang [15]. They presented a hybrid ant system algorithm to solve dynamic facility layout problem. Another research was a hybrid ant system algorithm for solving TSP in which ant colony, genetic algorithm, and simulated annealing are hybridized [16]. For the hybrid ant colony system (ACS), many researches were conducted including the work by Huang and Liao [17], Yoshikawa and Otani [18], Xing et al. [19], Liao et al. [20], Lin et al. [21], Hajipour et al. [22], and Min et al. [16]. The research by Katagiri et al. [23] is an example for hybrid MAX-MIN Ant System. To solve TSP problems, several hybrid ACO variants with other metaheuristic algorithms such as SA, PSO, ACO, ABC, and ANN were proposed. Bontoux and Feillet [24] proposed a hybrid ACO algorithm with local search procedures to solve TSP. Tsai et al. [25] presented a hybrid ACO called ACOMAC algorithm for solving TSP. Beam-ACO algorithm is a hybrid ACO with beam search for solving TSP [26]. Chen and Chien presented a hybrid algorithm, called the genetic simulated annealing ant colony system with particle swarm optimization techniques, for solving TSP [27]. Junqiang and Aijia proposed a hybrid ant colony algorithm (HACO), which combined ACO with delete-cross to overcome the shortcoming of slow convergence speed of ACO [28]. Dong et al. [29] proposed an algorithm, called cooperative genetic ant system (CGAS) for solving TSP, which hybridized both GA and ACO to improve the performance of ACO. Recently, Gündüz et al. [30] presented a hybrid ACO with ABC for solving TSP. In addition, Mahi et al. [31] proposed a new algorithm in which ACO was hybridized with PSO and 3-Opt for solving small TSP instances. The PSO was used to determine the optimum values of the two main parameters of ACO which affected algorithm performance and the 3-Opt was used to escape from the local optima found by ACO algorithm. Furthermore, Yousefikhoshbakht et al. [32] proposed REACSGA for solving small TSP instances which employed the modified ACS for generating initial diversified solutions and GA for intensification mechanisms.

As noted above, previous studies show that ACO still has drawbacks. The performance of these studies was dramatically decreased when dealing with large-scale instances. To the best of my knowledge, no research has been done to hybridize elitist ant system with SA, mutation, and local search. Therefore, in this research a new hybrid elitist ant system with SA, mutation operator, and local search procedure is introduced for solving TSP. Introducing SA can help ACO to escape from the local optima. On the other hand, determining initial solution of SA is almost difficult. Therefore, the use of the ACO is useful in the generation of SA initial solution. While introducing the mutation operation to ACO algorithm will enhance the algorithm performance, expand the diversity of population, and inhibit the premature convergence. Applying either SA or mutation is based on the diversity level of the population. After applying SA or mutation, elitist ant system goes through a local search procedure to speed up the convergence.

The rest of the paper is structured as follows.  Section 2 presents the TSP formulation.  Section 3 describes the hybrid algorithm. The experimental results are presented in Section 4. Conclusions and future work are given in Section 5.

## 2. Traveling Salesman Problem

TSP is an active field of research in computer science. It demonstrates all the aspects of combinatorial optimization and comes under the set of NP-hard problems which cannot be solved optimally in a polynomial time [33]. Solving TSPs is an important part of applications in many practical problems within daily live [2–4].

TSP is represented as a connected graph *G*, consisting of a set of vertices *V*, an edges set *E*, and a set of distances *d* associated with each edge in *E* and stored in a distance matrix *D*. The value *d*_*ij*_ is the cost of traversing from vertex *i* ∈ *V* to vertex *j* ∈ *V* and the diagonal elements *d*_*ii*_ are zeros. Given this information, a tour in TSP is formulated as a cyclic permutation, called Hamiltonian cycle of *G* visiting each vertex in the graph exactly once, *π* of {1,2,…, *n*}, where *π*(*i*) is the city, on the tour, following city (*i*). The aim in solving TSP is to find a permutation *π* that minimizes the length of the tour as shown in

(1) minimize ∑i=1ndiπi.

It is worth mentioning that the total number of possible distinct feasible routes covering all cities *n* is (*n* − 1)!/2. This produced a problem which is very hard to solve (NP-hard problem).

## 3. Algorithm Design

An overview of the ACO, SA, mutation operator, and the proposed algorithm is presented in the following subsections.

### 3.1. Ant Colony Optimization

ACO is a population-based metaheuristic algorithm which was inspired by the foraging behavior of the real ants when searching for the shortest path from the food source to their nest. Analogically, the artificial ants search for good solutions iteratively in several generation. In each generation, every ant constructs its feasible solution path step by step guided by a transition rule that is a function of artificial pheromone and distance between two cities (heuristic information) [34] as shown in (2). After that, the ant lays down an amount of pheromone trail on the edges of its completed tour. In the next generation, ants are attracted by the pheromone trail. Therefore, this will guide the search in the search space towards good quality solutions.

TSP is identical to the foraging behaviors of real ants in nature. Therefore, applying ant colony optimization to solve TSP will be very simple. Equation (2) is used to calculate the probability of selecting city *j* by ant *k* to be visited after city *i*.

(2) $$\begin{array}{c} P_{ij}^{k}=\frac{\tau_{ij}^{\alpha }\eta_{ij}^{\beta }}{\sum_{j\in N_{i}^{k}}^{}\tau_{ij}^{\alpha }\eta_{ij}^{\beta }}\;\text{if }j\in N_{i}^{k}, \end{array}$$

where *τ*_*ij*_ denotes the amount of pheromone between city *i* and city *j*, *η*_*ij*_ indicates the distance between city *i* and city *j* (priori available heuristic information), *β* is the parameter that represents the relative importance of the pheromone (dynamic evaluation) versus the heuristic value (static evaluation), and *N*_*i*_^*k*^ is a set of cities which ant *k* has not yet visited. Therefore, the selection probability is proportional to the product of the static and dynamic evaluation.

In the dynamic evaluation, two pheromone update rules are used to calculate the amount of pheromone on each edge between cities. The first rule is called the local update rule as shown in

(3) $$\begin{array}{c} \tau_{ij}\left(\;t+1\;\right)=\left(\;1-\rho \;\right)\tau_{ij}\left(\;t\;\right)+\overset{m}{\underset{k=1}{\sum }}\Delta \tau_{ij}^{k}\left(\;t\;\right), \end{array}$$

where 0 < *ρ* ≤ 1 is the pheromone trail evaporation rate in local update rule and *m* is the number of ants. Thus, the local update rule is decreasing the pheromone trails by a constant factor (pheromone evaporation). The second rule is the global update rule which adds extra amount pheromone trail to the best route in the population. It is worth mentioning that the best route is the shortest route as in elitist strategy [10], the extended version of original ant system algorithm. Equation (4) shows the definition of the global update rule in elitist ant system:

(4) $$\begin{array}{c} \Delta \tau_{ij}^{gb}\left(\;t\;\right)=\left\{\begin{array}{cc} \frac{e}{L^{gb}\left(t\right)} & \text{if edge}\left(i,j\right)\in T^{gb}\\ 0 & \text{otherwise}, \end{array}\right. \end{array}$$

where *T* ^gb^ is the best route, *L* ^gb^(*t*) is the distance of the best route, and *e* is a positive integer. This means that the edges belonging to the global-best tour get an additional amount of pheromone each time the pheromone is updated.

The pseudocode of the basic ACO is illustrated in Algorithm 1.

### 3.2. Simulated Annealing

SA is a trajectory-based optimization technique. It is basically an iterative improvement strategy with a criterion that accepts higher cost configurations sometimes. The first attempt to apply SA for solving the combinatorial optimization problems was in the 80s of the last century [35, 36]. An overview of simulated annealing, its theoretical development, and application domains is shown in [9]. Simulated annealing was inspired by physical annealing process of solids in which a solid is first heated and then cooled down slowly to reach a lower state of energy. Metropolis acceptance criterion [37], which models how thermodynamic systems moves from one state to another state, is used to determine whether the current solution is accepted or rejected.

The original Metropolis acceptance criterion was that the initial state of a thermodynamic system was chosen at energy *G* and temperature *T*. Holding *T* constant, the initial configuration of the system is perturbed to produce new configuration and the change in energy Δ*G* is calculated. The new configuration is accepted unconditionally if Δ*G* is negative whereas it is accepted if Δ*G* is positive with a probability given by the Boltzmann factor shown in (5) to avoid trapping in the local optima:

(5) $$\begin{array}{c} {exp}^{-\Delta Cost/Temperature}. \end{array}$$

This processes is then repeated until reaching a good sampling statistics for the current temperature, and then the temperature is decreased and the process is repeated until a frozen state (free energy state) is reached at *T* = 0. The analogy between the states of a physical system and optimization problems is given as follows: (i) the current configuration of the thermodynamic system is similar to the current solution of the optimization problem; (ii) the thermodynamic system energy is similar to the objective function of optimization problem; and (iii) ground status of the thermodynamic system is similar to the global minimum of the optimization problem.  Algorithm 2 shows the general structure of SA.

### 3.3. Mutation Operator

Mutation operator is inspired from the evolutionary algorithms in which each ant in the population will be given the chance to be altered based on a predefined probability. This operator may help the ant colony algorithm to explore new areas in the search space. It can be applied by randomly exchanging the position of two cities in the tour which leads to generate a new solution that is not very far from the original one.  Algorithm 3 shows the main steps of the mutation operation.

### 3.4. The Proposed Algorithm

In this section, a new algorithm called annealing elitist ant system with mutation operator and local search for solving the traveling salesman problem is introduced. Given *n* cities in TSP instance, first, the elitist ant system will generate the initial population with *m* ants and each ant will randomly choose a city as its starting city. The pheromone intensity level between any two cities is initialized with small positive constant *s*_0_. The iteration counter, which will record the number of iterations the proposed algorithm executes so far, is initially set to zero. For every interval *i* during the execution of the algorithm, where *i* is a predefined number, the elitist ant system will call either simulated annealing or mutation operation, based on the diversity of the elitist ant system, to enhance the algorithm performance. If the diversity is greater than 0.5, the algorithm needs intensification which can be achieved by applying simulated annealing for ratio of the solutions pool. On the contrary, if the diversity is less than 0.5, it means that the algorithm will lose the diversity and may get stuck in the local minima. Therefore, the ant algorithm needs to increase the diversity by applying the mutation operator with a predefined probability. The diversity between the fitness of the ants in the population was measured by calculating the Euclidean Distance (ED) as shown in

(6) $$\begin{array}{c} ED=\frac{\bar{d}-d_{min}}{d_{max}-d_{min}}, \end{array}$$

where $\bar{d}$ is the average distance between the fitness of the best ant and the fitness of the remaining ants in the population. *d*_min_ and *d*_max_ are the distances of the worst ant fitness and the second best ant fitness from the fitness of best ant respectively [38]. ED has a range of values between 0 and 1. If ED is low, most ants in the population are concentrating around the best ant and so the convergence is achieved. If ED is high, most of the ants are not biased towards the current best ant. Therefore, ED gives a description for the population variation and the lack of similarity between ants. The following are the steps of the proposed algorithm.

**Step 1 .**

After the initial stage mentioned above, each ant in the population builds its tour by applying the transition rule followed by the local pheromone update rule:

(1) *Transition Rule*. The *i*th ant decides the next city *j* to be visited according to (2).
(2) *Local Pheromone Update Rule*. After all ants complete their tours, the local update rule of the pheromone trails is applied for each route according to (3).

**Step 2 .**

Calculate the route cost of each ant. After that, apply the global pheromone update rule in which the amount of pheromone is added to the best route which has the lowest cost. This rule is defined in (4).

**Step 3 .**

Calculate the diversity of the population. If the diversity is high, the algorithm needs intensification by applying SA. Otherwise, the algorithm needs to regain the diversity by applying the mutation operator.

*Simulated Annealing*. If the diversity value is greater than 0.5, then the elitist ant system will use SA technique to enhance the selected ant by encoding its tour reached by the ant system into SA. Here, the tour of the selected ant is encoded as the initial state of SA. Initialize the system temperature to *T*_0_. In SA, two bits in the ant *A* are randomly selected for exchange to generate a new mutate ant *A*′ and its energy (cost) is evaluated accordingly. If the energy of ant *A*′ is better than that of ant *A* or a random number generated between 0 and 1 is less than the Boltzmann factor as defined by (5), then the new ant *A*′ is accepted. Otherwise, the ant *A* remains unchanged. Reduce the temperature by annealing schedule or by factor 0.0 < *α* < 1.0. Iteratively, this process will be repeated until *T*_0_ reaches to a predefined low temperature. If tour path of ant *A*′ is better than that of *A*, then the enhanced ant *A*′ is included in the ants population. Otherwise, the ant *A* remains unchanged and is placed back into the ants population.

*Mutation Operator*. To maintain the diversity of the proposed algorithm, the mutation operator is introduced for further exploration of new areas of the search space. If the diversity value is less than 0.5, then the elitist ant system will use the mutation operation to enhance the selected ant. In this process, an ant is selected randomly with a predefined probability. If a random number between 0 and 1 is less than the predefined mutation rate, then the algorithm selects an ant to mutate. Two bits in the ant *A* are selected randomly for exchange to generate a new mutate ant *A*′ and calculate its tour.

**Step 4 .**

Apply the local search procedure for further enhancement.

**Step 5 .**

Again, apply the global pheromone update rule according to (4).

**Step 6 .**

**Figure 1:**
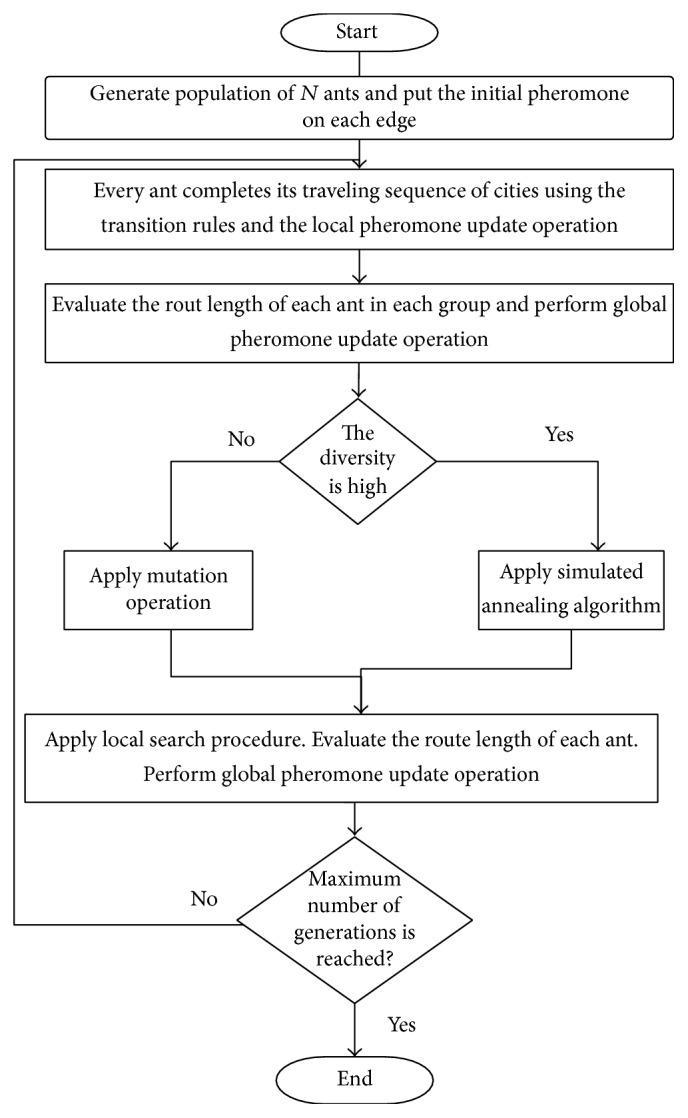
The flowchart of the proposed method algorithm.

If the termination condition is satisfied, then return the best route with its length. Otherwise, go to Step 1.  Figure 1 shows the flowchart of the proposed algorithm.

## 4. Experimental Results and Discussion

The experimental results to examine the validity and the performance of the proposed algorithm were introduced in this section. The experiments were conducted using 24 TSP standard benchmark problems, with different length, from TSPLIB [39, 40]. The proposed algorithm has been implemented in Java on an Intel Core-i7 PC. The Object Oriented Paradigm (OOP) and different data structures have been used to optimize its code. All experiments were conducted on the symmetric TSP.

As in other metaheuristic algorithms, the quality of the solutions created by the proposed algorithm was affected largely by the different values of the parameters. Thus, a number of different alternative values were examined to tune the parameters of the proposed algorithm.  Table 1 provides the parameters which show variations in the range of values while default values of other parameters were taken. Finally, the selected parameter values are those that achieved the best computational results with respect to the quality of the solution. All of the parameter values have been determined by the experiments on Eil51, lin318, and fl1400 TSP instances which represent small, medium, and large instances, respectively. In these experiments, the proposed algorithm was stopped when reaching the optimal solution or 1000 iterations. The optimum combinations of the parameters are shown in Table 1. Afterward, the algorithm was initialized with a population of 25 ants using *α* = 1 and *β* = 5 which control the influence of pheromone trail and heuristic information (edge cost) in selection of the next city by transition rule. The parameter *q*_0_ was set to 0.05 which specifies the intensification/diversification rate. The initial value of the pheromone trail was set to be *τ*_0_ = 0.5 and the maximum number of iteration was 1000 iterations.

All of the instances included in TSPLIB have already been examined in the literature and their optimality results can be used to compare algorithms according to the best and the average values. Different instances with different size were selected. These instances can be classified into three groups based on their lengths. The first group is the smallest group which includes 8 instances varying in length between 51 and 100 cities. The second is the medium group which includes 10 instances varying in length between 101 and 318 cities. The third is the large-scale group which includes 5 instances with length between 575 and 1655 cities. Therefore, the results were collected after conducting the experiments 10 times for each instance and took the best results, the average results, and the standard deviation for comparison with previous work. Two different experiments were conducted for the evaluation. Both of them were configured according to the parameters setting as shown in Table 1.

In the first experiment, the proposed algorithm was compared with the basic elitist ant system algorithm (EAS). Six evaluation measures were used to evaluate both algorithms. These measures are best solution, worst solution, average solution, standard deviation, number of iterations, and running time of algorithms.  Table 2 shows the superiority of the proposed algorithm over EAS in computational results for all evaluation measures. As can be seen from the tabulated values, the quality of the solutions obtained by the proposed algorithm was significantly better than the solutions obtained by EAS. This superiority of the proposed algorithm may be attributed to the introduction of SA which exploited the detected promising solutions to speed up the learning capability of the algorithm. Meanwhile, the addition of the mutation operator enhanced global search capability of the algorithm, prevented it from being trapped in local optima, and thus improved its performance. Moreover, the introduction of the local search strategy increased the speed of algorithm convergence.

**Figure 2:**
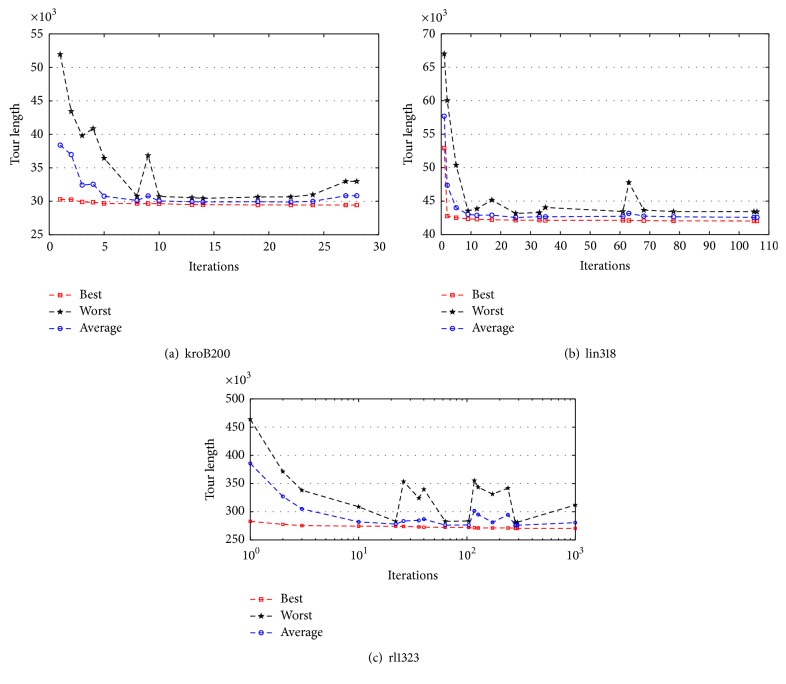
The performance of the proposed algorithm with three TSP instances representing short, medium, and large instances.

Figure 2 shows the behavior of the proposed algorithm with three TSP instances. They were chosen to represent short, medium, and large instances, and as such, the findings can be generalized to the other instances.

Figure 2(a) shows the results from a single run for korB200 instance. In this instance, the proposed algorithm reached the optimal solution that is 29437 in all ten runs. This figure depicts best, average, and worst solution obtained during the run. Strong optimization capability of proposed algorithm could be inferred. Diversified solutions, high convergence speed, and stagnation avoidance can be observed from the convergence behavior of the algorithm. Specifically, the figure shows how quickly the optimal solution, that is 29437, was found after 26 iterations. The trend goes down fast towards the optimum solution in the early iterations until it approaches the optimum solution. It is clear that no stagnation happened during the search process as observed from the figure.

Figure 2(b) illustrates the results from a single run of the proposed algorithm for the lin318 instance. In nine out of ten runs, the proposed algorithm reached the optimal solution that is 42029. This figure presents the graph which shows the convergence behavior of the algorithm. It is clearly noticed that the proposed algorithm has high convergence speed with diversified solutions. The algorithm succeeded in avoiding the potential stagnation and premature convergence as can be observed from the convergence behavior of the algorithm. According to the figure, there is no stagnation happened during the search process. Additionally, the algorithm converged to the best solution after a maximum of 106 iterations. The search space for this instance is medium although the algorithm has no problem in quickly finding the optimum solution.

Figure 2(c) demonstrates a single run for rl1323 instance containing 1323 cities. This graph plots the convergence behavior of the proposed algorithm over 1000 iterations. The algorithm reached a solution with cost near to optimal solution at iteration number 293 and never once changed afterwards. As it can be seen from the figure, in the initial stage, the diversity was high due to the variation of the population. However, as fitness function decreased, the diversity also decreased until the suboptimal solution was attained that is 270388. This smooth convergence was due to the good balance between diversification and intensification that proposed algorithm could provide. Although the search space for that instance was large, no stagnation happened during the search process.

**Figure 3:**
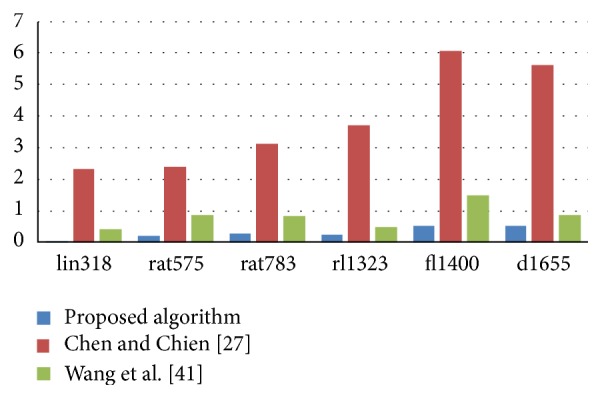
Percentage deviations of the average solution to the best known solution of the large-scale TSP instances for the proposed algorithm and the other algorithms.

In the second experiment, the proposed algorithm was compared with four state-of-the-art metaheuristic algorithms: Chen and Chien [27], Wang et al. [41], Yousefikhoshbakht et al. [32], and Mahi et al. [31]. The authors of these algorithms proved that their algorithms outperformed the other algorithms in the literature. The best found solution, the average one over all runs, the standard deviation, the percentage deviation of the best results, and the percentage deviation of the average results were used as evaluation measures for the comparison. The results are presented in Tables 3 and 4 and Figure 3.

In Tables 3 and 4, column 1 shows the TSP instances, column 2 shows the best known solutions, column 3 shows the algorithms, column 4 shows the best solutions over all runs, column 5 shows the average solution of all runs, column 6 presents the standard deviation, column 7 reveals the percentage deviation of the best results (PD_Best) compared to those of the best known solution, and column 8 reveals the percentage deviation of the average of the best solution of all runs (PD_Avg) in comparison to the best known solution. PD_Best was calculated by (7) and PD_Avg was calculated by (8).

(7) PD_Best=bestsolution−bestknownsolutionbestknownsolution×100,

(8) PD_Avg=avgsolution−bestknownsolutionbestknownsolution×100.

In Table 3, the proposed algorithm was compared with Chen and Chien [27] and Wang et al. [41] on 24 benchmark instances with cities from 51 to 1655. As can be seen in Table 3, for the 24 TSP instances, the proposed algorithm was much better than both algorithms on all medium and large instances, such as lin318, rat575, rat783, rl1323, fl1400, and d1655, with respect to the five evaluation measures mentioned above. There was no significant difference between the proposed algorithm, Chen and Chien [27] and Wang et al. [41] on the small instances with cities less than or equal to 100, with respect to best found solution and PD_Best.

In Table 4, the proposed algorithm was compared with Yousefikhoshbakht et al. [32] and Mahi et al. [31] on 15 and 8 benchmark instances, respectively, with cities from 51 to 200 as reported in their studies. As can be seen in Table 4, the values of columns best and PD_Best show that there was no significant difference between the proposed algorithm and Yousefikhoshbakht et al. [32] on the small instances with cities less than or equal to 100. For the larger instances, the proposed algorithm gained much better results than Yousefikhoshbakht et al. [32]. Comparing with Mahi et al. [31], the proposed algorithm achieved better results in all the 8 instances with respect to best found solution, average solution, PD_Best, and PD_Avg.

Figure 3 shows a comparison of the proposed algorithm to Chen and Chien [27] and Wang et al. [41] based on the percentage deviations of the average solution to the best known solution. It is clear that the proposed algorithm significantly gained smaller percentage deviations than Chen and Chien [27] and Wang et al. [41] in the large-scale TSP instances that is lin318, rat575, rat783, rl1323, fl1400, and d1655.

In summary, numerical results show that the proposed algorithm was effective. It was able to solve small and large size instances better than the existing algorithms. This is because of the proposed algorithm capability of searching the optimal solution until the last iterations without stagnation or premature convergence, especially for the medium and large TSP instances, compared to the other algorithms. In general, the results indicate that the structure of the proposed algorithm, which depends on the concepts of embedding simulated annealing, mutation operation, and local search procedure, achieved the balance between diversification and intensification and enabled algorithm to escape from local optima and speed up the convergence. These gave the proposed algorithm the superiority over the other algorithms in reaching the suboptimal/optimal solutions for TSP problems.

## 5. Conclusion

In this paper, a new hybridized metaheuristic algorithm, called annealing elitist ant system with mutation operator for traveling salesman problem, has been introduced. Experiments were conducted using 24 data sets obtained from the TSPLIB and the experimental findings of the proposed algorithm were compared with different state-of-the-art algorithms. The results illustrate that the proposed algorithm outperforms other algorithms and has smaller percentage deviations in comparison to Chen and Chien [27], Wang et al. [41], Yousefikhoshbakht et al. [32], and Mahi et al. [31] algorithms. For future work, the proposed hybrid algorithm can be enhanced by using adaptive parameter on-the-fly or tuning using fuzzy logic. In addition, further evaluation of the performance of the proposed hybrid algorithm can be done using asymmetric TSP. For generalization, the proposed algorithm can be applied for different optimization problems. Another enhancement can be introduced by implementing a parallel version of this algorithm.

## Figures and Tables

**Algorithm 1:**
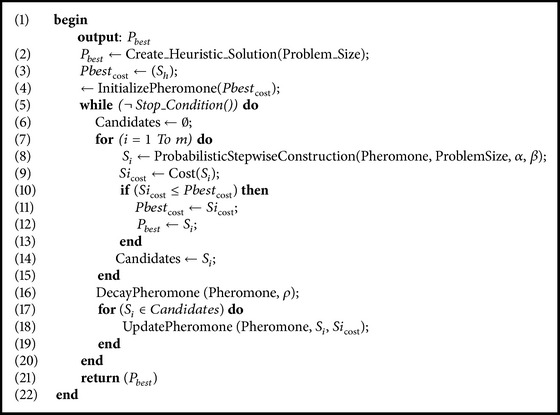
Pseudocode for ant system.

**Algorithm 2:**
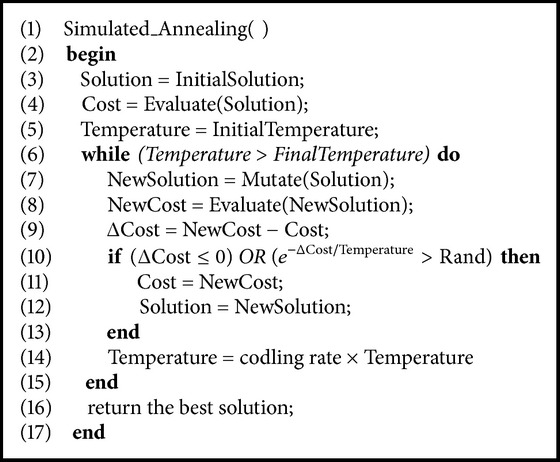
Simulated annealing.

**Algorithm 3:**
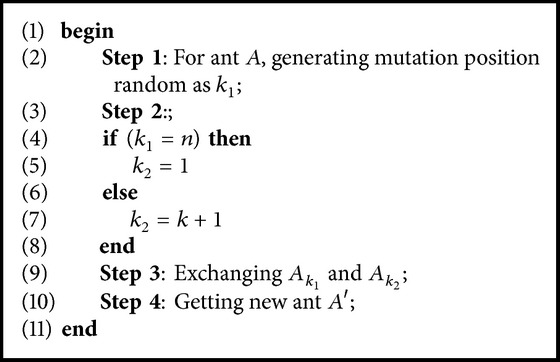
The pseudocode for applying the mutation operator for TSP problem.

**Table 1:** Parameter setting of the proposed algorithm.

Proposed algorithm parameters	Tested values	Optimum value
*α*	1 2 3 4 5	1 or 2
*α*	1 3 5 7 9	5 7 9
*ρ*	0.01 0.05 0.1 0.5 0.7	0.1 or less
*q*_0_	0.01 0.05 0.1 0.5 0.7	0.05
Number of ants	25 50 75 100	25
*Q *	50 100 500	100
SA Temp.	100 1000 5000 10000	1000
SA Alpha	0.5 0.7 0.9 0.99	0.99
Mutation rate	0.001 0.01 0.1 0.5	0.1

**Table 2:** Results obtained by the EAS and the proposed algorithm for the test problems according to the best solution, worst solution, average solution, standard deviation, number of iterations, and running time of algorithms.

Instance	Opt.	Method	Best	Worst	Average	Std. dev.	Iteration	Time (s)
eil51	426	EAS	430	474	442.3	14.38	999	0.01
		Proposed	**426**	**449**	**426**	**0.00**	**2**	**0.00**
eil76	538	EAS	547	653	563.9	13.25	999	0.02
		Proposed	**538**	**566**	**538**	**0.00**	**2**	**0.01**
eil101	629	EAS	661	778	677.1	9.27	999	0.04
		Proposed	**629**	**667**	**629**	**0.00**	**3**	**0.02**
berlin52	7542	EAS	7633	10674	7816.9	141.35	999	0.01
		Proposed	**7542**	**10804**	**7542**	**0.00**	**3**	**0.06**
bier127	118282	EAS	121978	151016	125064.1	1866.12	999	0.07
		Proposed	**118282**	**153876**	**118282**	**0.00**	**4**	**0.05**
ch130	6110	EAS	6386	7757	6515.3	83.80	999	0.07
		Proposed	**6110**	**6519**	**6110**	**0.00**	**4**	**0.05**
ch150	6528	EAS	6734	7771	6865.5	101.49	999	0.07
		Proposed	**6528**	**6937**	**6528**	**0.00**	**3**	**0.03**
rd100	7910	EAS	8240	10161	8422.2	151.46	999	0.04
		Proposed	**7910**	**8540**	**7910**	**0.00**	**2**	**0.01**
lin105	14379	EAS	14756	18010	15102.3	278.80	999	0.02
		Proposed	**14379**	**15315**	**14379**	**0.00**	**2**	**0.01**
lin318	42029	EAS	44981	58151	46293.6	917.35	999	1.42
		Proposed	**42029**	**43715**	**42042.4**	**42.37**	**12**	**0.54**
kroA100	21282	EAS	22085	29227	22603.8	450.09	999	0.04
		Proposed	**21282**	**23228**	**21282**	**0.00**	**2**	**0.01**
kroA150	26524	EAS	27560	31282	28370	504.96	999	0.12
		Proposed	**26524**	**27793**	**26524**	**0.00**	**4**	**0.05**
kroA200	29368	EAS	31499	41604	31886.1	249.14	999	0.11
		Proposed	**29368**	**30829**	**29368**	**0.00**	**9**	**0.20**
krob100	22141	EAS	22652	27908	23134.8	281.92	999	0.04
		Proposed	**22141**	**28127**	**22141**	**0.00**	**2**	**0.01**
krob150	26130	EAS	27248	34550	28099.5	512.53	999	0.14
		Proposed	**26130**	**27556**	**26130**	**0.00**	**2**	**0.02**
krob200	29437	EAS	31054	38684	32019	559.76	999	0.23
		Proposed	**29437**	**31024**	**29437**	**0.00**	**11**	**0.24**
kroc100	20749	EAS	21194	22485	21777.8	348.02	999	0.03
		Proposed	**20749**	**26834**	**20749**	**0.00**	**2**	**0.01**
krod100	21294	EAS	22205	29101	22872.5	457.97	999	0.03
		Proposed	**21294**	**22627**	**21294**	**0.00**	**3**	**0.02**
kroe100	22068	EAS	22699	31216	23460.4	486.84	999	0.07
		Proposed	**22068**	**23078**	**22068**	**0.00**	**2**	**0.01**
rat575	6773	EAS	7365	8398	7436.6	72.57	999	9.11
		Proposed	**6777**	**6986**	**6787.1**	**10.13**	**999**	**46.23**
rat785	8806	EAS	9706	12596	9860.8	128.84	999	26.79
		Proposed	**8811**	**9086**	**8829.7**	**15.02**	**999**	**66.85**
rl1323	270199	EAS	297599	392473	304626.9	5122.59	999	110.29
		Proposed	**270309**	**287323**	**270841.7**	**403.43**	**999**	**194.62**
fl1400	20127	EAS	22432	43829	23669.5	820.80	999	93.12
		Proposed	**20194**	**29176**	**20233.4**	**26.01**	**999**	**34.24**
d1655	62128	EAS	68182	92988	71629.3	1905.70	999	33.58
		Proposed	**62291**	**68922**	**62457.5**	**90.39**	**999**	**199.58**

**Table 3:** A comparison of the proposed algorithm with Chen and Chien [27] and Wang et al. [41] according to the best solution, average solution, standard deviation, the percentage deviation of the average solution (PD_Avg), and the percentage deviation of the best solution (PD_Best) found by the algorithms.

Instance	Opt.	Method	Best	Average	Std. dev.	PD_Best	PD_Avg
eil51	426	Proposed algorithm	**426**	**426**	**0.000**	**0.000**	**0.000**
		Chen and Chien [27]	427	427.27	0.450	0.235	0.298
		Wang et al. [41]	**426**	**426**	N/A	**0.000**	**0.000**
eil76	538	Proposed algorithm	**538**	**538**	**0.000**	**0.000**	**0.000**
		Chen and Chien [27]	**538**	540.2	2.940	**0.000**	0.409
		Wang et al. [41]	**538**	**538**	N/A	**0.000**	**0.000**
eil101	629	Proposed algorithm	**629**	**629**	**0.000**	**0.000**	**0.000**
		Chen and Chien [27]	630	635.23	3.590	0.159	0.990
		Wang et al. [41]	**629**	**629**	N/A	**0.000**	**0.000**
berlin52	7542	Proposed algorithm	**7542**	**7542**	**0.000**	**0.000**	**0.000**
		Chen and Chien [27]	**7542**	**7542**	**0.000**	**0.000**	**0.000**
		Wang et al. [41]	**7542**	**7542**	N/A	**0.000**	**0.000**
bier127	118282	Proposed algorithm	**118282**	**118282**	**0.000**	**0.000**	**0.000**
		Chen and Chien [27]	**118282**	119421.8	580.830	**0.000**	0.964
		Wang et al. [41]	**118282**	**118282**		**0.000**	**0.000**
ch130	6110	Proposed algorithm	**6110**	**6110**	**0.000**	**0.000**	**0.000**
		Chen and Chien [27]	6141	6205.63	43.700	0.507	1.565
		Wang et al. [41]	**6110**	6112.4		**0.000**	0.039
ch150	6528	Proposed algorithm	**6528**	**6528**	**0.000**	**0.000**	**0.000**
		Chen and Chien [27]	**6528**	6563.7	22.450	**0.000**	0.547
		Wang et al. [41]	**6528**	6531.84	N/A	**0.000**	0.059
rd100	7910	Proposed algorithm	**7910**	**7910**	0.000	**0.000**	**0.000**
		Chen and Chien [27]	**7910**	7987.57	62.060	**0.000**	0.981
		Wang et al. [41]	**7910**	**7910**	N/A	**0.000**	**0.000**
lin105	14379	Proposed algorithm	**14379**	**14379**	**0.000**	**0.000**	**0.000**
		Chen and Chien [27]	**14379**	14406.37	37.280	**0.000**	0.190
		Wang et al. [41]	**14379**	14379	N/A	**0.000**	**0.000**
lin318	42029	Proposed algorithm	**42029**	**42042.4**	**42.375**	**0.000**	**0.032**
		Chen and Chien [27]	42487	43002.9	307.510	1.090	2.317
		Wang et al. [41]	42081	42204.16	N/A	0.124	0.417
kroA100	21282	Proposed algorithm	**21282**	**21282**	**0.000**	**0.000**	**0.000**
		Chen and Chien [27]	**21282**	21370.47	123.360	**0.000**	0.416
		Wang et al. [41]	**21282**	21284.24	N/A	**0.000**	0.011
kroA150	26524	Proposed algorithm	**26524**	**26524**	**0.000**	**0.000**	**0.000**
		Chen and Chien [27]	**26524**	26899.2	133.020	**0.000**	1.415
		Wang et al. [41]	**26524**	26528.12	N/A	**0.000**	0.016
kroA200	29368	Proposed algorithm	**29368**	**29368**	**0.000**	**0.000**	**0.000**
		Chen and Chien [27]	29383	29738.73	356.070	0.051	1.262
		Wang et al. [41]	**29368**	29374.84	N/A	**0.000**	0.023
kroB100	22141	Proposed algorithm	**22141**	**22141**	**0.000**	**0.000**	**0.000**
		Chen and Chien [27]	**22141**	22282.87	183.990	**0.000**	0.641
		Wang et al. [41]	**22141**	22186.28	N/A	**0.000**	0.205
kroB150	26130	Proposed algorithm	**26130**	**26130**	**0.000**	**0.000**	**0.000**
		Chen and Chien [27]	**26130**	26448.33	266.760	**0.000**	1.218
		Wang et al. [41]	**26130**	26133.2	N/A	**0.000**	0.012
kroB200	29437	Proposed algorithm	**29437**	**29437**	**0.000**	**0.000**	**0.000**
		Chen and Chien [27]	29541	30035.23	357.480	0.353	2.032
		Wang et al. [41]	**29437**	29439.64	N/A	**0.000**	0.009
kroC100	20749	Proposed algorithm	**20749**	**20749**	**0.000**	**0.000**	**0.000**
		Chen and Chien [27]	**20749**	20878.97	158.640	**0.000**	0.626
		Wang et al. [41]	**20749**	20749	N/A	**0.000**	**0.000**
kroD100	21294	Proposed algorithm	**21294**	**21294**	**0.000**	**0.000**	**0.000**
		Chen and Chien [27]	21309	21620.47	226.600	0.070	1.533
		Wang et al. [41]	**21294**	21297.2	N/A	**0.000**	0.015
kroE100	22068	Proposed algorithm	**22068**	**22068**	**0.000**	**0.000**	**0.000**
		Chen and Chien [27]	**22068**	22183.47	103.320	**0.000**	0.523
		Wang et al. [41]	**22068**	22075.52	N/A	**0.000**	0.034
rat575	6773	Proposed algorithm	**6777**	**6787.1**	**10.126**	**0.059**	**0.208**
		Chen and Chien [27]	6891	6933.87	27.620	1.742	2.375
		Wang et al. [41]	6807	6830.88	N/A	0.502	0.855
rat783	8806	Proposed algorithm	**8811**	**8829.7**	**15.019**	**0.057**	**0.269**
		Chen and Chien [27]	8988	9079.23	52.690	2.067	3.103
		Wang et al. [41]	8859	8877.92	N/A	0.602	0.817
rl1323	270199	Proposed algorithm	**270309**	**270841.7**	**403.434**	**0.041**	**0.238**
		Chen and Chien [27]	277642	280181.5	1761.660	2.755	3.694
		Wang et al. [41]	270919	271481.6	N/A	0.266	0.475
fl1400	20127	Proposed algorithm	**20194**	**20233.4**	**26.014**	**0.333**	**0.529**
		Chen and Chien [27]	20593	21349.63	527.880	2.315	6.075
		Wang et al. [41]	20314	20428.48	N/A	0.929	1.498
d1655	62128	Proposed algorithm	**62291**	**62457.5**	**90.388**	**0.262**	**0.530**
		Chen and Chien [27]	64151	65621.13	1031.940	3.256	5.622
		Wang et al. [41]	62463	62670.52	N/A	0.539	0.873

**Table 4:** A comparison of the proposed algorithm with Yousefikhoshbakht et al. [32] and Mahi et al. [31] according to the best solution, average solution, standard deviation, the percentage deviation of the average solution (PD_Avg), and the percentage deviation of the best solution (PD_Best) found by the algorithms.

Instance	Opt.	Method	Best	Average	Std. dev.	PD_Best	PD_Avg
eil51	426	Proposed algorithm	**426**	**426**	**0.000**	**0.000**	**0.000**
		Yousefikhoshbakht et al. [32]	**426**	N/A	N/A	0.000	N/A
		Mahi et al. [31]	N/A	426.45	0.610	N/A	0.106
eil76	538	Proposed algorithm	**538**	**538**	**0.000**	**0.000**	**0.000**
		Yousefikhoshbakht et al. [32]	**538**	N/A	N/A	**0.000**	N/A
		Mahi et al. [31]	N/A	538.3	0.470	N/A	0.056
eil101	629	Proposed algorithm	**629**	**629**	**0.000**	**0.000**	**0.000**
		Yousefikhoshbakht et al. [32]	**629**	N/A	N/A	0.000	N/A
		Mahi et al. [31]	N/A	632.7	2.120	N/A	0.588
berlin52	7542	Proposed algorithm	**7542**	**7542**	**0.000**	**0.000**	**0.000**
		Yousefikhoshbakht et al. [32]	**7542**	N/A	N/A	0.000	N/A
		Mahi et al. [31]	N/A	7543.2	2.370	N/A	0.016
ch150	6528	Proposed algorithm	**6528**	**6528**	**0.000**	**0.000**	**0.000**
		Mahi et al. [31]	N/A	6563.95	27.580	N/A	0.551
lin105	14379	Proposed algorithm	**14379**	**14379**	**0.000**	**0.000**	**0.000**
		Yousefikhoshbakht et al. [32]	**14379**	N/A	N/A	**0.000**	N/A
		Mahi et al. [31]	N/A	14379.15	0.480	N/A	0.001
lin318	42029	Proposed algorithm	**42029**	**42042.4**	**42.375**	**0.000**	**0.032**
		Yousefikhoshbakht et al. [32]	42543	N/A	N/A	1.223	N/A
kroA100	21282	Proposed algorithm	**21282**	**21282**	**0.000**	**0.000**	**0.000**
		Yousefikhoshbakht et al. [32]	**21282**	N/A	N/A	**0.000**	N/A
		Mahi et al. [31]	N/A	21445.1	78.240	N/A	0.766
kroA150	26524	Proposed algorithm	**26524**	**26524**	**0.000**	**0.000**	**0.000**
		Yousefikhoshbakht et al. [32]	26611	N/A	N/A	0.328	N/A
kroA200	29368	Proposed algorithm	**29368**	**29368**	**0.000**	**0.000**	**0.000**
		Yousefikhoshbakht et al. [32]	**29368**	N/A	N/A	**0.000**	N/A
		Mahi et al. [31]	N/A	29646.05	114.710	N/A	0.947
kroB100	22141	Proposed algorithm	**22141**	**22141**	**0.000**	**0.000**	**0.000**
		Yousefikhoshbakht et al. [32]	**22141**	N/A	N/A	**0.000**	N/A
kroB150	26130	Proposed algorithm	**26130**	**26130**	**0.000**	**0.000**	**0.000**
		Yousefikhoshbakht et al. [32]	26202	N/A	N/A	0.276	N/A
kroB200	29437	Proposed algorithm	**29437**	**29437**	**0.000**	**0.000**	**0.000**
		Yousefikhoshbakht et al. [32]	29509	N/A	N/A	0.245	N/A
kroC100	20749	Proposed algorithm	**20749**	**20749**	**0.000**	**0.000**	**0.000**
		Yousefikhoshbakht et al. [32]	20754	N/A	N/A	0.024	N/A
kroD100	21294	Proposed algorithm	**21294**	**21294**	**0.000**	**0.000**	**0.000**
		Yousefikhoshbakht et al. [32]	21335	N/A	N/A	0.193	N/A
kroE100	22068	Proposed algorithm	**22068**	**22068**	**0.000**	**0.000**	**0.000**
		Yousefikhoshbakht et al. [32]	**22068**	N/A	N/A	**0.000**	N/A
